# Brain-Wide Activations Related to Forepaw Force Control Identified by Behaving Mouse FMRI

**DOI:** 10.1523/ENEURO.0106-26.2026

**Published:** 2026-08-18

**Authors:** Vishwas Jindal, Jason Veizaj, Zoë Schuler, Erika de Jesus, Daniel W. Wesson, David Vaillancourt, Shahabeddin Vahdat

**Affiliations:** ^1^Department of Applied Physiology and Kinesiology, University of Florida, Gainesville, Florida 32608; ^2^McKnight Brain Institute, University of Florida, Gainesville, Florida 32610; ^3^McLean Hospital, Harvard Medical School, Belmont, Massachusetts 02478; ^4^Department of Pharmacology & Therapeutics, University of Florida, Gainesville, Florida 32610; ^5^Department of Bioengineering, University of California Riverside, Riverside, California 92507

**Keywords:** brainstem, fMRI, forelimb control, mouse, sensorimotor cortex, skill learning

## Abstract

Control of upper limb force is crucial for motor skill acquisition. Rodent models have been instrumental in elucidating the behavioral and neural mechanisms underlying skilled movements. Integrating these models with advanced neuroimaging approaches, such as awake functional magnetic resonance imaging (fMRI) in behaving mice, enables whole-brain mapping of motor activity. However, experimental paradigms supporting awake fMRI during upper limb motor behavior in mice remain limited. Here, we developed an MRI-compatible head–fixation system that enables male and female mice to perform a unilateral forepaw force control task for water reward during ultrahigh-field (11.1 T) fMRI. Mice successfully acquired the task, as evidenced by increased rewarded presses, convergence of force output toward the rewarded threshold, and reduced force variability. Significant activation clusters related to forepaw force were identified across multiple cortical regions, including the primary and secondary motor cortex, anterior cingulate cortex, and primary somatosensory cortex. Activation extended to subcortical structures, including the cerebellum, striatum, hypothalamus, and thalamus (ventrolateral and ventroposterior nuclei). Analysis of limb kinematics from synchronized video recordings revealed strong spatial correspondence between forepaw-related and force-dependent activation maps. Region-of-interest analyses further identified engagement of medullary structures, specifically the lateral rostral medulla and caudal medulla (CauM), in forepaw force control. Notably, the cerebellum and CauM exhibited later peak responses relative to cortical regions. Together, these results establish a robust framework for awake fMRI during forelimb motor tasks and provide a comprehensive map of cortical, subcortical, and brainstem circuits underlying forelimb force control in mice.

## Significance Statement

Control of forelimb force is fundamental to motor function, yet its underlying neural mechanisms remain unclear. We present an awake mouse fMRI approach that enables simultaneous measurement of forepaw force and whole-brain activity during a controlled motor task. This method reveals a distributed network spanning cortical, subcortical, cerebellar, and brainstem regions involved in forelimb force control. By directly linking behavior to brain-wide activation, this framework overcomes key limitations of anesthetized imaging and provides a translational bridge between animal and human studies. Importantly, this framework can serve as a bridge between animal and human studies and can be used to investigate circuit-level dysfunction in disorders such as stroke and to develop targeted strategies for motor recovery.

## Introduction

Control of applied force is fundamental to motor function in daily activities, and its disruption following neurological conditions such as stroke significantly impairs quality of life. Human neuroimaging studies using functional magnetic resonance imaging (fMRI) have identified a distributed network of cortical regions involved in forelimb and handgrip force control, including the anterior cingulate cortex (ACC), primary motor cortex (M1), premotor cortex, and primary somatosensory cortex (S1; Ehrsson et al., 2001; Kuhtz-Buschbeck et al., 2001; Bernard et al., 2002; Sterr et al., 2009; Coombes et al., 2011; Mizuguchi et al., 2014; Andrushko et al., 2021; Karabanov et al., 2023), along with subcortical structures such as the basal ganglia, cerebellum, and thalamus (Spraker et al., 2007, 2012; Tanaka et al., 2009; Wasson et al., 2010). These regions contribute to error correction, regulation of movement rate, and modulation of force output based on task demands (Vaillancourt et al., 2007; Coombes et al., 2011; Grafton and Tunik, 2011). Notably, cerebellar activity increases during slower force rates, whereas premotor cortex activity is elevated during faster force rates (Tanaka et al., 2009). Subcortical structures such as the dorsal striatum (dSTR) and ventral thalamus further regulate movement amplitude and velocity (Vaillancourt et al., 2007; Coombes et al., 2011; Grafton and Tunik, 2011). Despite these advances, human studies remain largely correlational and provide limited insight into underlying causal mechanisms.

Rodent studies have provided mechanistic insights into neural circuits underlying force control using electrophysiology, optogenetics, and behavioral paradigms. For example, S1 inhibition abolishes motor adaptation to force (Mathis et al., 2017), while pathway-specific cortical perturbations differentially affect movement direction and amplitude (Park et al., 2022). Ventral tegmental area activity correlates with continuous head and body forces measured using a head-fixation system in mice (Hughes et al., 2020). In the brainstem, silencing neurons in the caudal medulla (CauM) impairs skilled reaching, and the lateral rostral medulla (Lat-RM), which projects to CauM, coordinates diverse forelimb actions through distinct neuronal populations (Esposito et al., 2014; Ruder et al., 2021). Behavioral studies further show that lesions to M1 or the corticospinal tract impair forelimb strength and dexterity, often leading to compensatory reliance on alternative pathways such as the reticulospinal tract (Farr and Whishaw, 2002; Welch et al., 2009; Hays et al., 2013; García-Alías et al., 2015). Despite these advances, brain-wide activation patterns underlying forelimb movements and force regulation in rodents remain largely unexplored.

Functional MRI in mice provides a noninvasive, translational approach to bridge this gap by enabling whole-brain imaging. However, the need for anesthesia to stabilize animals and to suppress head motion during scanning in the majority of mouse fMRI studies has limited its applications for studying behavior in rodents (Ferris et al., 2006; Tsurugizawa et al., 2020). Awake fMRI has been used to examine sensory processing, optogenetic stimulation, and operant behaviors, revealing widespread cortical and subcortical activation (Desai et al., 2011; Harris et al., 2015; Liang et al., 2015; Chang et al., 2016; Yoshida et al., 2016; Madularu et al., 2017; Desjardins et al., 2019; Han et al., 2019; Chen et al., 2020; Gutierrez-Barragan et al., 2022; Zeng et al., 2022; Hike et al., 2023; Poplawsky et al., 2023). Notably, only one study has examined awake mouse fMRI during a motor task, demonstrating activation in sensorimotor and limbic regions during lever pressing (Fonseca et al., 2022). No study so far has used fMRI to investigate brain-wide correlates of limb force control in rodents.

To address this limitation, we developed an MR-compatible head–fixation system that enables fMRI during a motor task involving forepaw force control. This setup integrates force measurement via a sensitive MR-compatible sensor, upper limb kinematic tracking using an MR-compatible camera, and real-time water reward delivery while minimizing noise and motion artifacts. We hypothesized that forelimb force control engages a distributed network including contralateral sensorimotor cortices (M1, M2, S1, ACC), subcortical structures (thalamus, striatum), cerebellum, and brainstem nuclei such as the CauM and Lat-RM.

## Materials and Methods

### Animals

Adult male and female C57BL/6J mice (8–12 weeks old; *n* = 17, 13 males, 4 females) were purchased from the Jackson Laboratory and used for the experiment. Of these mice, 15 successfully reached criterion performance, and 2 mice were removed from analysis because their head implants fell off during the training. Animal experimental protocols and husbandry were approved by the Institutional Animal Care and Use Committee of the University of Florida. The mice were housed under environmentally controlled conditions with a 12 h reversed dark/light cycle, and food and water were provided *ad libitum*, before starting the water restriction as described below. Behavioral shaping and imaging were performed during the dark phase. Data used in this study were partially reported in Jindal et al. (2026), which focused on functional connectivity analysis of corticomedullary networks across humans and mice. Here, we apply a distinct analytical framework to address different research questions, focusing on behavioral improvements, activation maps linked to distinct task parameters, and regional characteristics of task-related activation, which were not examined in the prior study.

### Surgical procedure for head bar implantation

The mice underwent a surgical procedure on a stereotactic frame, during which an in-house designed 3D-printed head bar was implanted on the top of their skulls. This allowed for head fixation during both behavioral training and scanning sessions. The shape of the 3D-printed head bar was specifically designed to allow proper placement of the MRI radiofrequency (RF) saddle coil on top of the head covering the whole brain. Mice were anesthetized using isoflurane in pure oxygen and maintained at a level of 1.5–2% throughout the surgical procedure. Body temperature, heart rate, and respiration were monitored every 15 min to ensure they remained within the physiological range. Before surgery, the top of each mouse’s head was shaved and cleaned with saline and betadine. Once positioned in a stereotactic frame, a small round incision was made in the scalp, and the skin was carefully removed to expose the skull. The head bar was secured onto the skull using light-cured dental cement (CLEARFIL AP-X and CLEARFIL SE BOND, Kuraray Noritake Dental). After the surgery, mice were kept on a heating pad to maintain body temperature. Subcutaneous injections of buprenorphine (0.01 mg/kg) and 0.3 ml saline were administered following surgery to minimize discomfort and prevent dehydration. After recovering from anesthesia, the mice were placed in individual cages for housing and monitored for at least 3 d postsurgery.

### Water restriction

After a 1 week recovery period following surgery, mice underwent water restriction to ∼85% of baseline body weight to promote task engagement (Goltstein et al., 2018). Initially, the animals received 2 ml of water on the first day of water restriction, followed by 1.6 ml on the second day, 1.4 ml on the third day, 1.2 ml on the fourth day, and 1 ml on both the fifth and sixth days. On the seventh day, they received 0.8 ml of water. This reduced amount on the seventh day was provided to maintain the animals’ motivation for the subsequent day, which marked the beginning of the behavioral experiment.

### Behavioral experiment

#### Experimental setup

**Figure 1.**
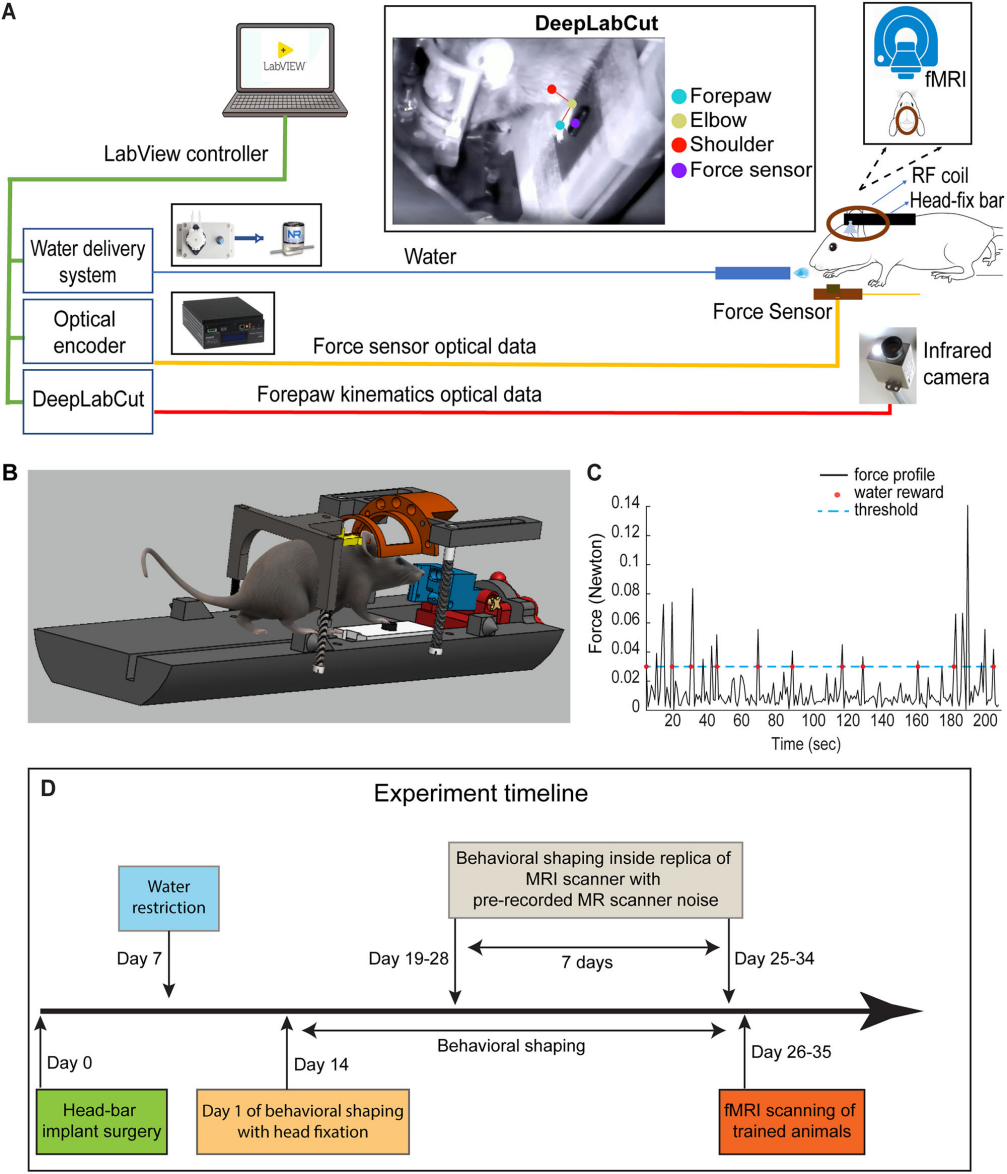
Experimental setup for awake mouse fMRI during a forelimb force control task. ***A***, Schematic of the forelimb force control system and its subunits, including DeepLabCut-based tracking of forepaw kinematics [adapted from Jindal et al. (2026)]. ***B***, Schematic of the MR-compatible sled for mouse head fixation. The figure shows the saddle-shaped transmit/receive MR coil (orange) and the force transducer accessible to the mouse via a button (black) placed in front of the right forepaw (Jindal et al., 2026). ***C***, Representative forepaw press force profile recorded during fMRI, with red dots indicating water reward delivery after successful presses exceeding 0.03 N (dashed line). ***D***, Schematic of the training paradigm, MRI scanning, and surgical procedures.

The forepaw force control experiment utilized a 3D-printed sled equipped with a force sensor, head fixture, RF coil fixture, and a water delivery station (Fig. 1). The sled, designed using SolidWorks and 3D printed in standard resin material, allowed for head fixation of the mouse during the task. A miniature force transducer, designed in-house for precise force measurement (0.005 N resolution), was integrated into the setup. Force measurements were transmitted via optical fiber through an optical encoder (Hyperion si155, Luna Innovations) for digitization and were sent to a laptop. An infrared MR-compatible camera (12M-i, MRC Systems) was mounted on the sled to monitor and record forelimb movements via a mirror. The water delivery system consisted of a 3D-printed spout connected via 0.0625-inch-inner-diameter tubing to a 12 V pinch valve (NResearch, 225P011-21) linked to a water supply. All components were interfaced through a LabJack data acquisition system and controlled using a custom LabVIEW program, which delivered water when the applied force exceeded a predefined threshold.

Behavioral experiments were conducted in a darkened environment under dim red light or complete darkness. To acclimate mice to the scanner environment, a scaled-down replica of the MRI scanner was constructed in the behavioral room, and the sled containing the mice was placed inside it. During the 7 d preceding MRI scans, prerecorded scanner noise matched in intensity to the actual scanner was played during daily training sessions to habituate animals to the MRI environment and minimize motion during subsequent imaging sessions.

#### Head-fixed motor–learning task

Mice were introduced to a 3D-printed sled and underwent head fixation before being trained in a water-reinforced forepaw force control task. Animals learned to press a stationary force sensor (≥0.03 N) to receive water rewards (0.004 ml). Initial shaping involved experimenter-assisted placement of the forepaw on the sensor, with mice typically acquiring the association within 1–2 d. Subsequently, animals performed the task independently, with each successful press followed by an intertrial interval (ITI) that was progressively increased from 2 to 12 s. Presses during the ITI were not rewarded. Force signals were processed in LabVIEW using a moving average with automatic baseline correction due to sustained forepaw contact or body weight. Successful presses were defined as force changes exceeding 0.03 N. Daily training sessions progressively increased in duration up to 40 min or until 200 rewards were obtained (0.8 ml), with total daily water intake limited to 1 ml. Training continued until mice achieved >100 successful presses at a 12 s ITI (12–21 d, *n* = 15), with additional days included as needed for scheduling. Notably, withholding presses during the ITI was not part of the training criterion but was used to prevent continuous pressing during scanning.

During the final 7 d, session duration was extended to 50 min, with the first 10 min unrewarded to acclimate animals to the scanning setup. Sessions with no presses for >15 min were terminated and repeated the following day. Learning criteria were based on the number of successful presses rather than ITI compliance. Behavioral data were recorded using LabVIEW, including force output and reward delivery. Performance metrics included mean force per press, force variability, interpress interval, total number of rewarded presses, total number of presses, and total number of unrewarded presses. Learning-related changes were assessed by comparing the first and last 3 d of training using paired *t* tests with false discovery rate correction (*p* < 0.05).

### MRI data acquisition

All fMRI data were acquired using an 11.1 T Bruker scanner at the Advanced Magnetic Resonance Imaging and Spectroscopy Facility (AMRIS), McKnight Brain Institute, University of Florida. Behavioral data were recorded using LabVIEW, along with video recordings, consistent with the training setup, and scanner triggers were logged for synchronization. Each animal underwent a standardized scanning session with identical setup and imaging protocols. An MRI saddle linear coil (AMRIS/National MagLab) was used for RF transmission and reception. Body temperature was maintained via circulating warm water. A localizer scan was first acquired to verify head positioning at the magnet center, followed by manual adjustment as needed. Magnetic field homogeneity was optimized using shimming over an ellipsoidal volume encompassing the brain and brainstem.

Subsequently, a low-resolution structural image was captured using a T2-weighted spin echo (SE) sequence using the following parameters: repetition time (TR), 6 s; echo time (TE), 33 ms; bandwidth, 36.0577 kilohertz (kHz); number of slices, 27; and resolution, 0.346 × 0.346 × 0.5 mm^3^. Furthermore, a high-resolution structural T2-weighted MR scan was acquired using a multislice T2-weighted fast SE sequence signal, using the following parameters: TR, 7 s; TE, 36 ms; bandwidth, 100 kHz; number of slices, 41; and resolution, 0.098 × 0.098 × 0.35 mm^3^. The fMRI scans were then acquired using SE echoplanar imaging (EPI) to record the blood oxygenation level-dependent (BOLD) signal, using the following parameters: coronal slices, TR, 2 s; TE, 15 ms; bandwidth, 326.087 kHz; number of slices, 27; number of volumes, 210; and resolution, 0.346 × 0.346 × 0.5 mm^3^.

Due to RF coil positioning, full brain coverage was not achieved in six animals, resulting in partial brainstem coverage. Accordingly, brainstem analyses were restricted to nine animals, while cortical and subcortical analyses included all 15 animals. Each fMRI run lasted 7 min and was repeated 3–5 times depending on task performance. Runs were considered valid if animals produced ≥15 presses, with 2–3 valid runs per animal (53 ± 7.1 presses per run, mean ± SEM). During scanning, animals performed the trained task while behavior was monitored via an MR-compatible camera. Scans were paused if irregular force signals or signs of distress were observed and resumed after a 10 min break.

### Neuroimage data analysis

fMRI data were analyzed using FSL (Smith et al., 2004). Preprocessing included skull stripping of functional and structural images, with manual brain masks drawn for each animal. For each fMRI run, a reference volume was selected, and brain masks were dilated and applied to isolate the brain tissue. All runs within each animal were aligned using 6 df rigid-body transformations (FSL).

A slice-wise nonlinear transformation (ANTs) was computed to register the reference fMRI run to the corresponding low-resolution T2–weighted image, acquired with matched slice orientation and resolution. This nonlinear transformation was used to register the EPI data to the nondistorted T2-weighted image, correcting susceptibility-induced distortions in the EPI data. Low- and high-resolution T2–weighted images were then aligned using rigid-body registration. A representative high-resolution T2–weighted image was selected as the template for group-level analysis, and each animal’s structural image was registered to this template using a 12-parameter affine transformation (FSL). These transformations were concatenated to map functional data to template space. Atlas-based regions of interest (ROIs) from the Allen Mouse Brain Atlas (CCFv3; Oh et al., 2014) were registered to the template space and visually inspected, with adjustments made as needed to ensure accurate alignment. For brainstem analyses, the left and right Lat-RM ROIs contained 548 and 545 voxels, respectively; the left and right medial rostral medulla (MRM) ROIs contained 274 and 277 voxels, respectively, and the left and right CauM ROIs contained 184 and 196 voxels, respectively.

Functional data preprocessing further included motion correction, high-pass temporal filtering (*σ* = 60 s), and spatial smoothing (Gaussian kernel, 0.7 mm FWHM; Vahdat et al., 2021). For each run, the recorded force time-series was low-pass filtered (20 Hz) and downsampled to match the fMRI sampling rate (TR, 2 s). The force regressor was generated from the continuous force signal recorded throughout the scan and therefore included both rewarded and nonrewarded presses. The force and reward time-series were convolved with a double-gamma hemodynamic response function and used as regressors of interest in a general linear model (GLM). Temporal derivatives were included to account for timing variability, and six motion parameters (translations and rotations) were added as nuisance regressors. Motion outliers identified using fsl_motion_outliers were also included in the model.

Mid-level fixed-effects analyses were performed to average activation maps across runs (2–3 per animal). Group-level statistical analysis was conducted using mixed-effects modeling with FILM and local autocorrelation correction (Woolrich et al., 2004). Resulting *Z* statistic maps were thresholded at *Z* > 2.3 and corrected for multiple comparisons using Gaussian random field (GRF) theory (cluster-level *p* < 0.05, corrected). Notably, GLM analyses used the continuous force signal to identify brain regions whose activity covaried with changes in applied force.

ROI-based analyses were performed by extracting mean activation values from atlas-defined regions. In addition, BOLD percent signal change relative to baseline was computed by extracting mean time-series from active voxels (*p* < 0.05, uncorrected) and deconvolving the force signal (34 s window). Finally, separate regressors for rewarded and unrewarded presses were included to dissociate motor-related from reward-related neural activity.

### DeepLabCut motion analysis

Forepaw, elbow, and shoulder positions were tracked using DeepLabCut (version 2.3.11; Nath et al., 2019; Lauer et al., 2022). A total of 10 frames from each of 35 videos were manually labeled, with 95% of the data used for training. A ResNet-50–based network was trained using default parameters for 100,000 iterations. Model performance was evaluated using one shuffle, yielding a test error of 5.33 pixels and a training error of 1.76 pixels (image size, 720 × 480 pixels). Predicted coordinates were filtered using a likelihood threshold (*p*-cutoff = 0.6) and used for subsequent analysis. The trained network was then applied to videos from similar experimental sessions. The resulting kinematic time-series were downsampled to match the fMRI acquisition rate (TR = 2 s) and used as regressors in separate GLMs to identify brain regions whose activity covaried with each limb segment’s displacement. Mid-level and group-level GLM analyses followed the same pipeline described above to generate statistical maps of kinematics-related activation. To assess potential collinearity between force, reward, and kinematic regressors, pairwise coefficients of determination (*R*^2^) were calculated and are reported in Extended Data Figure 3-3.

## Results

### Learning-related improvements in forepaw force control

We devised a forelimb skill learning task in head-fixed, water-deprived mice by shaping them to press a force sensor with their right forepaw (Fig. 1). Animals received a water reward when the applied force exceeded 0.03 N. An ITI was progressively introduced and increased to 12 s to structure task performance and reduce continuous pressing. Training success was defined by the number of rewarded presses, emphasizing force control rather than press timing. On average, animals reached criterion in 17 ± 5 d (mean ± SD).

**Figure 2.**
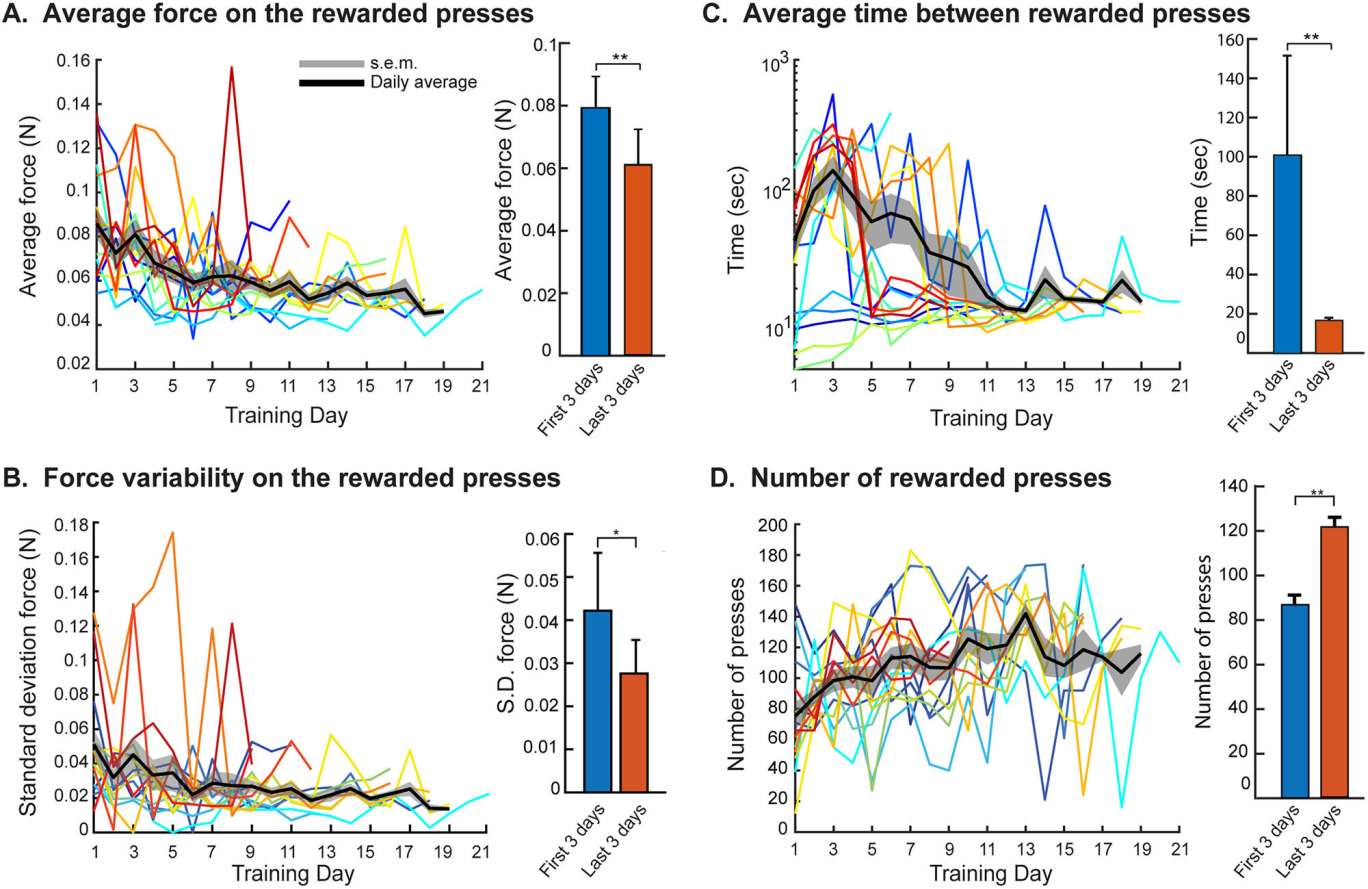
Enhancement of task performance reflected by increases in rewarded presses and forepaw force accuracy during training. ***A***, The average applied force on rewarded presses decreased and approached the force threshold as training progressed. Colored lines represent individual animals across training days, while the black line and gray shading denote mean ± SEM across 15 animals. Mice applied significantly less force on the transducer at the end of training (average of the last 3 d) compared with the beginning (first 3 d). ***B***, The standard deviation of the force on rewarded press decreased over training, indicating improved consistency in force control. ***C***, As learning progressed, the average time between rewarded press decreased, reflecting increased motivation and task engagement. ***D***, The number of rewarded presses increased significantly over the course of training. Individual colors represent different mice and are consistent across panels. Blue bars represent averages from the first 3 d, and orange bars represent averages from the last 3 d. Error bars indicate SEM (paired-sample *t* test, *p < 0.05; **p < 0.005). Additional behavioral metrics are shown in Extended Data Figure 2-1.

Using this task, we observed progressive refinement in forepaw force control across training, reflected by changes in both the mean and variability of the applied force (Fig. 2). Compared with early training (first 3 d), the average force during late training (last 3 d) decreased toward the reward threshold (0.03 N; *p* = 0.003, paired two-tailed *t* test, corrected; Fig. 2*A*). Force variability was also significantly reduced during late training compared with early training (*p* = 0.02, corrected; Fig. 2*B*).

10.1523/ENEURO.0106-26.2026.f2-1 Figure 2-1 **Behavioral metrics related to press timing during training. (A)** The mean ratio of the interval between rewarded presses to the inter-trial interval (ITI) decreased significantly over the course of training, indicating improved temporal precision and task learning. **(B)** The average time between each press remained stable across training days. **(C)** The total number of presses increased significantly during training, reflecting increased task engagement and performance. **(D)** The total number of unrewarded presses also increased across training, consistent with an overall increase in pressing behavior as animals became more engaged in the task. Colored lines represent individual animals, the black line represents the daily group average, and the gray shaded region indicates ± s.e.m. Bar plots show mean ± s.e.m. values averaged across the first 3 and last 3 training days (**p < 0.005, paired sample t-test). Display conventions are as in Figure 2. Download Figure 2-1, TIF file.

Mice initially exhibited freezing behavior and low motivation to press, but this improved over the course of training, resulting in a reduction in the average interval between rewarded presses (Fig. 2*C*). This interval decreased significantly from early to late training, approaching the imposed ITI (12 s; *p* = 0.003, corrected; Fig. 2*C*), and was accompanied by an increase in the number of rewarded presses (*p* < 0.005, corrected; Fig. 2*D*).

The total number of presses also increased across training, from 175 ± 27 presses during the first 3 d to 530 ± 56 presses during the last 3 d (mean ± SEM; Extended Data Fig. 2-1*C*). Similarly, the number of unrewarded presses increased from 139 ± 18 to 411 ± 54 presses (Extended Data Fig. 2-1*D*), consistent with increased task engagement as animals acquired the task. To assess timing adaptation, we compared the interval between rewarded presses to the imposed ITI (Extended Data Fig. 2-1*A*). Although the ratio between these intervals decreased significantly across training (early, 32.30; late, 1.56; *p* = 0.0007, corrected), it did not converge to one, indicating that animals did not consistently withhold presses during the ITI. This was further supported by the increase in unrewarded presses across training (Extended Data Fig. 2-1*D*). Consistently, the average interval between all presses remained unchanged across sessions (*p* > 0.05; Extended Data Fig. 2-1*B*), suggesting that training primarily improved force control and task engagement rather than precise timing.

### Brain-wide activation maps related to forepaw force control

Having reached criterion performance, 15 animals underwent awake fMRI scanning to measure whole-brain activity during the forepaw force control task. Movie 1 shows an example of an animal performing the task inside the scanner. Each animal contributed at least two functional runs (35 runs total). Head motion during scanning was minimal, with translational displacements below half a voxel in all directions and small rotational displacements (mean ± SD, *x* = 0.0351 ± 0.0068 mm; *y* = 0.0179 ± 0.0036 mm; *z* = 0.1519 ± 0.0189 mm; rotations, *x* = 0.0194 ± 0.0025 rad; *y* = 0.0136 ± 0.0019 rad; *z* = 0.0035 ± 0.0004 rad). The low levels of motion observed during imaging were facilitated by the custom head-fixation apparatus and the habituation procedures performed before scanning. A representative example of absolute and relative head displacement during an awake fMRI scan is shown in Extended Data Figure 3-2. Movie 2 shows representative raw and motion-corrected EPI images across the scanning session. Motion parameters were included as nuisance regressors in the GLM to account for residual motion-related effects.

**Movie 1.** Representative example of a head-fixed mouse performing the forepaw force control task inside the MRI scanner. The animal presses a force transducer with the right forepaw to receive a water reward upon exceeding the force threshold (0.03 N). [View online]

**Movie 2.** Representative raw and motion-corrected EPI images across a scanning session. Motion correction reduces image displacement artifacts, and residual motion parameters were included as nuisance regressors in the GLM. [View online]

**Figure 3.**
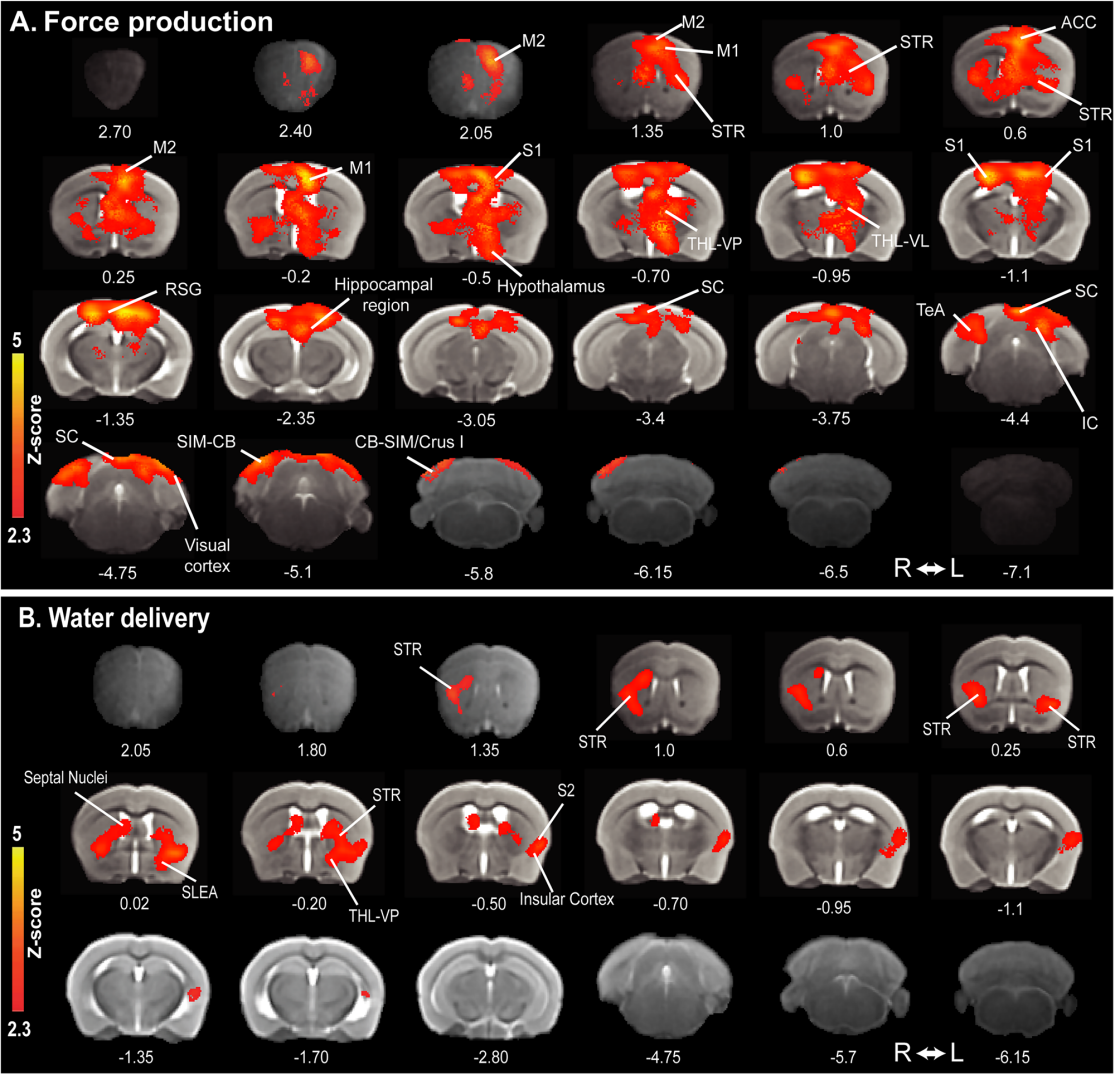
Group-level activation maps associated with forelimb force control and water reward delivery (*n* = 15 animals). ***A***, The activation map associated with the level of forepaw force showed significant activation clusters in various brain regions including cortical sensorimotor areas, dSTR, cerebellar cortex, and thalamus. Activation maps were generated using the continuous force regressor [a subset of these data was previously reported in the Supplementary Materials of Jindal et al. (2026)]. ***B***, On the other hand, ventral striatum, insular cortex, septal nuclei, thalamus, and amygdala were activated in association with water reward delivery. Group-level (*n* = 15) statistical maps are corrected for multiple comparisons using GRF (*Z* > 2.3; *p* < 0.05). Color bar indicates *Z* scores. The number below each slice indicates distance relative to the bregma. Activation maps show all slices containing significant clusters, overlaid on a high-resolution T2–weighted template image from a representative animal. Abbreviations: primary and motor cortex (M1), secondary motor cortex (M2), anterior cingulate cortex (ACC), primary somatosensory cortex (S1), secondary somatosensory cortex (S2), granular retrosplenial cortex (RSG), striatum (STR), thalamus ventral–posterior nucleus (THL-VP), thalamus ventral–lateral nucleus (THL-VL), superior colliculus (SC), inferior colliculus (IC), temporal association area (TeA), cerebellum simple lobule (CB-SIM). Activation maps during rewarded and nonrewarded presses are shown in Extended Data Figure 3-1. Representative head motion during awake fMRI is shown in Extended Data Figure 3-2. Collinearity between force, reward, and kinematic regressors is reported in Extended Data Figure 3-3.

Figure 3*A* shows the average brain activation associated with forepaw force (*Z* > 2.3; cluster-level *p* < 0.05; GRF-corrected). Significant cortical activation was observed in M1, M2, ACC, S1, and the granular retrosplenial cortex (RSG). Subcortical activation included the dSTR, hypothalamus, hippocampal regions, and thalamic nuclei (ventral–posterior, VP, and ventral–lateral, VL), as well as the superior and inferior colliculi and temporal association cortex. Cerebellar activation was evident in Crus1 and the simple lobule (SIM). Although activation was largely bilateral, sensorimotor cortices showed a modest contralateral bias relative to the active forepaw (compare Fig. 5).

10.1523/ENEURO.0106-26.2026.f3-1 Figure 3-1 **Group-level activation maps during rewarded and non-rewarded presses.** Activation maps show regions significantly engaged during rewarded (orange-yellow) and non-rewarded (blue) forepaw presses. Rewarded trials activated widespread cortical sensorimotor, striatal, thalamic, and hypothalamic regions, whereas non-rewarded trials recruited a more restricted network including S1, ACC, superior colliculus (SC), visual cortex, and cerebellar lobules. Group-level maps (n = 15) are corrected for multiple comparisons using GRF (Z > 2.3, p < 0.05). Abbreviations are as in Figure 3. All activation maps are shown at identical z-coordinates to Figure 3A. Download Figure 3-1, TIF file.

10.1523/ENEURO.0106-26.2026.f3-2 Figure 3-2 **Representative head motion during awake fMRI.** Absolute (blue) and relative (green) displacement estimates across a representative functional imaging run. Absolute displacement was calculated relative to the reference volume, whereas relative displacement reflects frame-to-frame motion between consecutive volumes. Download Figure 3-2, TIF file.

10.1523/ENEURO.0106-26.2026.f3-3 Figure 3-3 Correlation between the applied force (left columns) and water delivery (right columns) time course with movement kinematic time-series (forepaw, elbow, and shoulder displacement) derived from DeepLabCut. Values represent mean correlation coefficients (r ± SEM), mean coefficients of determination (R²), and associated statistical measures across animals. Although several correlations reached statistical significance, the low R² values indicate that force, reward, and kinematic regressors shared relatively little variance and captured distinct aspects of task performance. Download Figure 3-3, DOCX file.

**Figure 4.**
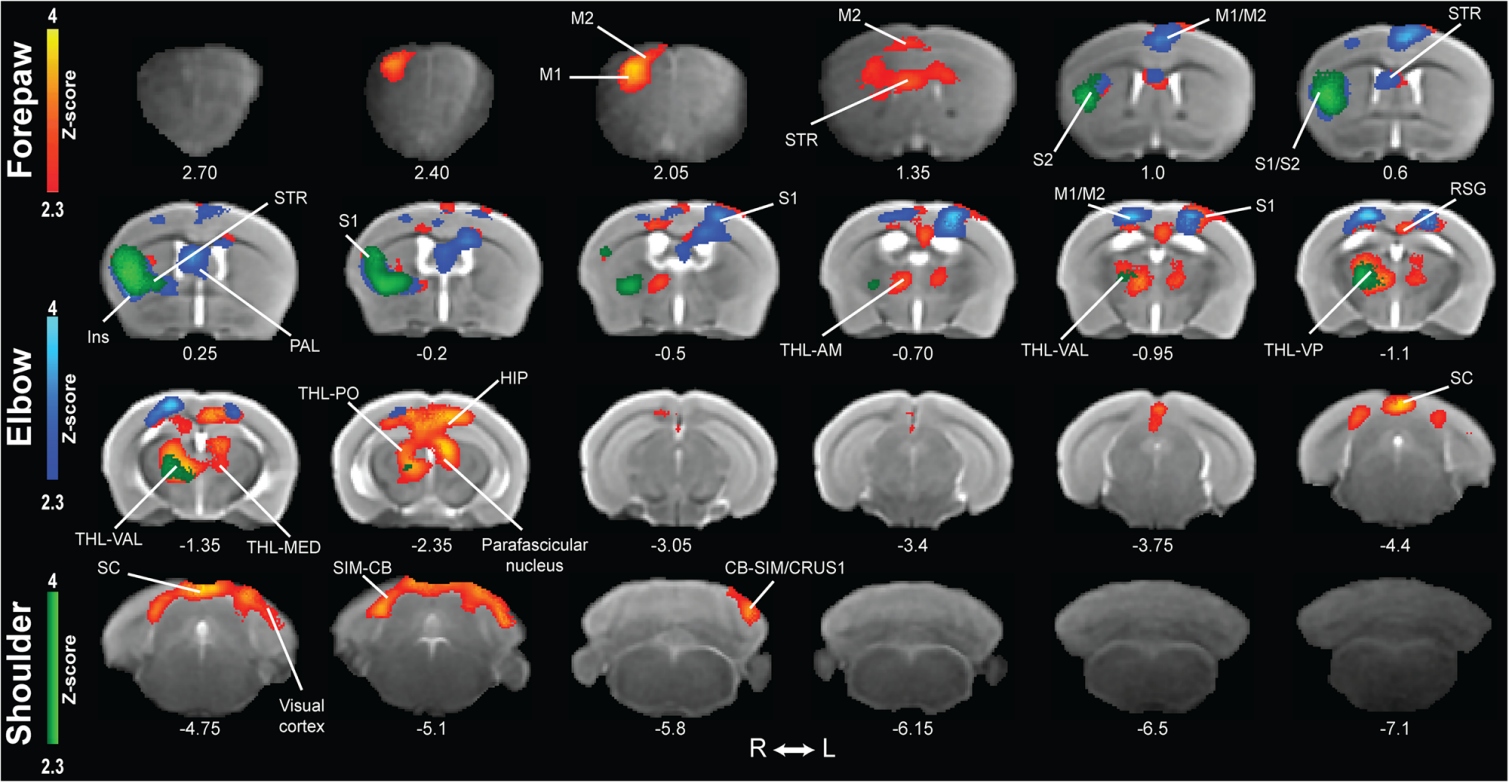
Group-level activation maps associated with forepaw, elbow, and shoulder displacement during task performance (*n* = 15 animals). Activation maps show regions preferentially associated with displacement of individual joints tracked using DeepLabCut: forepaw (red), elbow (blue), and shoulder (green). Distinct but partially overlapping representations are observed across cortical (M1, M2, S1, S2, Ins), striatal (STR), thalamic (THL-VAL, THL-VP, THL-PO, THL-VM, THL-MED), and cerebellar (SIM-CB, CRUS1) regions. Group-level statistical maps (*n* = 15) are corrected for multiple comparisons using GRF (*Z* > 2.3; *p* < 0.05). Abbreviations are as in Figure 3: THL-VM, ventromedial thalamic nucleus; THL-MED, medial thalamic nucleus. All activation maps are shown at identical *z*-coordinates to Figure 3*A*. Correlations between limb kinematics and head motion estimates for each animal are reported in Extended Data Figure 4-1.

10.1523/ENEURO.0106-26.2026.f4-1 Figure 4-1 Correlation between movement kinematics (forepaw, elbow, and shoulder displacement) derived from DeepLabCut and head-motion displacement estimates for each animal. Values represent correlation coefficients for individual animals, along with group mean ± SEM and associated statistics. Download Figure 4-1, DOCX file.

**Figure 5.**
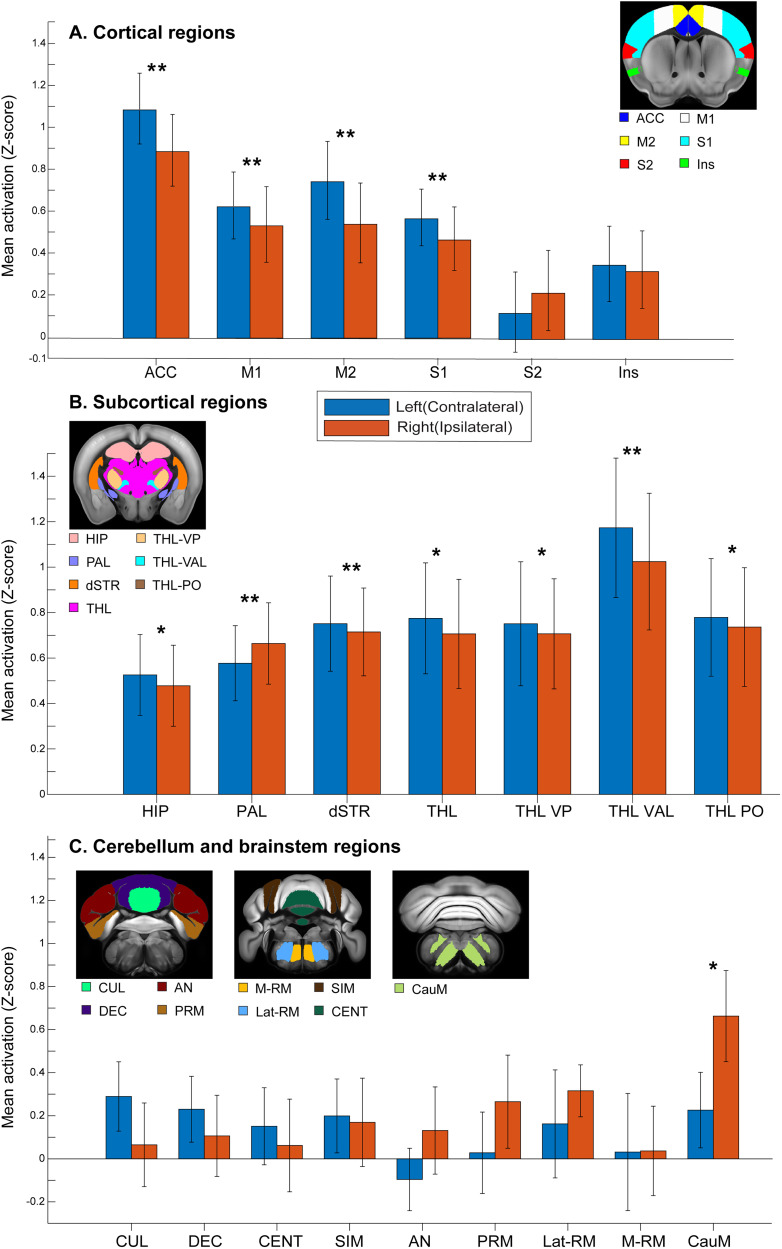
Task-based fMRI activation in key cortical, subcortical, and brainstem regions related to forepaw force control. Average force-related response (*Z* score) in the activated voxels in cortical sensorimotor regions (***A***), subcortical regions (***B***), and cerebellar and brainstem regions (***C***), on the left (blue, contralateral to the paw) and right (red, ipsilateral to the paw) sides. Cortical, subcortical, and cerebellar analyses included all animals (*n* = 15), whereas brainstem analyses were restricted to animals with complete brainstem coverage (*n* = 9). In the cortex, ACC, M1, M2, and S1 showed significant force-related activations bilaterally, with stronger activation on the contralateral side. In subcortical areas, HIP, PAL, dSTR, and three thalamic nuclei (VAL, PO, and VP) showed significant force-related activations. Contralateral VAL showed the strongest activation among thalamic nuclei. In the cerebellum, no subregions showed significant activation, although the contralateral CUL showed a trend toward significance. In the medulla, significant activation was observed in the ipsilateral CauM (uncorrected), whereas MRM did not show significant activation. Error bars indicate SEM (*p < 0.05; **p < 0.005) for cortical and subcortical analyses (multiple comparison corrected). Brainstem ROI analyses are shown uncorrected for multiple comparisons. Abbreviations are as in Figure 3, and dorsal striatum (dSTR), pallidum (PAL), hippocampus (HIP), insula (Ins), thalamus ventral–posterior nucleus (THL-VP), thalamus posterior nucleus (THL-PO), thalamus ventral–anterior–lateral nucleus (THL-VAL), culmen lobule (CUL), declive lobule (DEC), central lobule (CENT), simple lobule (SIM), ansiform lobule (AN), paramedian lobule (PRM), lateral rostral medulla (Lat-RM), medial rostral medulla (M-RM), caudal medulla (CauM). Individual animal data are shown in Extended Data Figure 5-1. Activation in control regions is shown in Extended Data Figure 5-2.

10.1523/ENEURO.0106-26.2026.f5-1 Figure 5-1 **Task-based fMRI activation in cortical, subcortical, cerebellar, and brainstem regions across individual animals.** Average force-related BOLD response (Z score) across left (blue, contralateral to the paw) and right (orange, ipsilateral) hemispheres for each animal in **(A)** cortical, **(B)** subcortical, and **(C)** cerebellar and brainstem regions. Each dot represents one animal, and lines connect paired hemispheric values within the same subject. Data are shown for cortical, subcortical, and cerebellar regions (*n* = 15 mice) and for brainstem regions (*n* = 9 mice). Download Figure 5-1, TIF file.

10.1523/ENEURO.0106-26.2026.f5-2 Figure 5-2 Average force-related activation (*Z* score) in the control regions, including the amygdala and the fourth ventricle. Display conventions are as in Figure 4. Download Figure 5-2, TIF file.

We next examined brain activation associated with water reward (Fig. 3*B*; *Z* > 2.3, cluster-level *p* < 0.05, corrected). Significant activation was observed in the secondary somatosensory cortex, insular cortex, piriform cortex, ventral striatum, sublenticular extended amygdala, septal nuclei, and THL–VP. No significant negative activations were detected for either forepaw force or reward.

To identify brain activation related to movement kinematics, we analyzed the displacement of upper limb landmarks (forepaw, elbow, and shoulder) from synchronized video during task performance using DeepLabCut. The resulting activation maps (Fig. 4; *Z* > 2.3, cluster-level *p* < 0.05, corrected) revealed distinct but partially overlapping representations. Activation associated with forepaw displacement (Fig. 4, orange) closely matched the force-related map (Fig. 3*A*), engaging cortical and subcortical regions including M1, M2, ACC, S1, S2, STR, RSG, insular cortex, thalamus, and hippocampus, with activation extending to the superior colliculus and cerebellum (Crus1, SIM). Elbow displacement (Fig. 4, blue) was associated with more bilateral activation centered in the sensorimotor cortex (M1, M2, S1), insula, pallidum, and dSTR, whereas shoulder displacement (Fig. 4, green) showed more restricted activation, primarily in the dSTR, thalamus, and insula. No significant hemispheric differences were observed in forepaw-related activation within primary sensorimotor cortices (M1, M2, S1; *p* > 0.05). Notably, correlations between limb kinematics and image-derived head motion were low and not significant across animals (*p* > 0.05), indicating minimal coupling between limb movement and head motion (Extended Data Fig. 4-1). Furthermore, collinearity between the applied force, water reward, and kinematic regressors was low, with mean coefficients of determination (*R*^2^) below 0.03 for all pairwise comparisons, indicating minimal shared variance among these regressors (Extended Data Fig. 3-3).

Comparing rewarded and nonrewarded presses (Extended Data Fig. 3-1) revealed that rewarded presses elicited widespread activation across cortical (M1, M2, ACC, RSG, S1) and subcortical regions (striatum, thalamus, hypothalamus), as well as the cerebellum. In contrast, nonrewarded presses produced weaker and more spatially restricted activation, primarily in S1, ACC, superior colliculus, visual cortex, and cerebellum.

### Region-specific activations across cortical, subcortical, and brainstem areas underlying forelimb force control

To quantify activation across brain regions, the average task-related activation within atlas-defined masks of key sensorimotor regions (Allen Mouse Brain Atlas; Oh et al., 2014) was computed for each animal. Two-way ANOVA models were used to assess the effects of region and laterality, and their interaction, on task-related activation. These analyses revealed a largely bilateral activation pattern across cortical and subcortical regions (Fig. 5*A*,*B*). For cortical regions, two-way repeated–measures ANOVA revealed a significant main effect of region (*p* = 2.7 × 10^−5^), with no effect of laterality (*p* = 0.3) and no interaction (*p* = 0.4). Post hoc analyses identified significant activation in ACC, M1, M2, and S1 (*p* < 0.05, corrected; Fig. 5*A*). For subcortical regions, two-way ANOVA showed a main effect of region (*p* = 0.01), with no effect of laterality (*p* = 0.65) and no interaction (*p* = 0.88). Significant activations were observed in the hippocampus, pallidum, dSTR, and thalamus (*p* < 0.05, corrected; Fig. 5*B*). Within the thalamus, three nuclei were significantly associated with forepaw force: ventral posterior (VP), ventral–anterior–lateral (VAL), and posterior (PO). Among these, VAL showed the strongest contralateral activation, with significant differences between VAL and VP (*p* = 0.009, corrected), but not between VAL and PO.

Figure 5*C* illustrates activation in cerebellar and brainstem subregions. Brainstem analyses were restricted to nine animals with full coverage, as coil positioning in six animals resulted in partial medullary coverage. In the cerebellum, two-way repeated–measures ANOVA revealed no significant effects of region (*p* = 0.74), laterality (*p* = 0.9), or interaction (*p* = 0.08). Similarly, no significant effects were observed in the brainstem (region, *p* = 0.29; laterality, *p* = 0.23; interaction, *p* = 0.10). Given the absence of interaction effects, regions were collapsed across laterality for post hoc testing. No cerebellar subregions showed significant activation (*p* > 0.05, corrected), although the culmen (CUL) showed a trend (*p* = 0.059). In contrast, significant activation was observed in the CauM (*p* = 0.03, one-sample *t* test, uncorrected), whereas neither Lat-RM nor MRM showed significant activation when activity was collapsed across hemispheres. Notably, brainstem activation was predominantly ipsilateral, consistent with prior anatomical reports (Ruder et al., 2021; Yang et al., 2023).

Extended Data Figure 5-1 shows task-related activation across regions in individual animals, demonstrating consistent hemispheric organization alongside intersubject and regional variability. We also calculated task-related activation in the amygdala and fourth ventricle as control regions. As expected, neither of these regions showed any task-related activation related to forelimb force control (Extended Data Fig. 5-2).

### Hemodynamic response time-locked to the forepaw press in key brain regions

**Figure 6.**
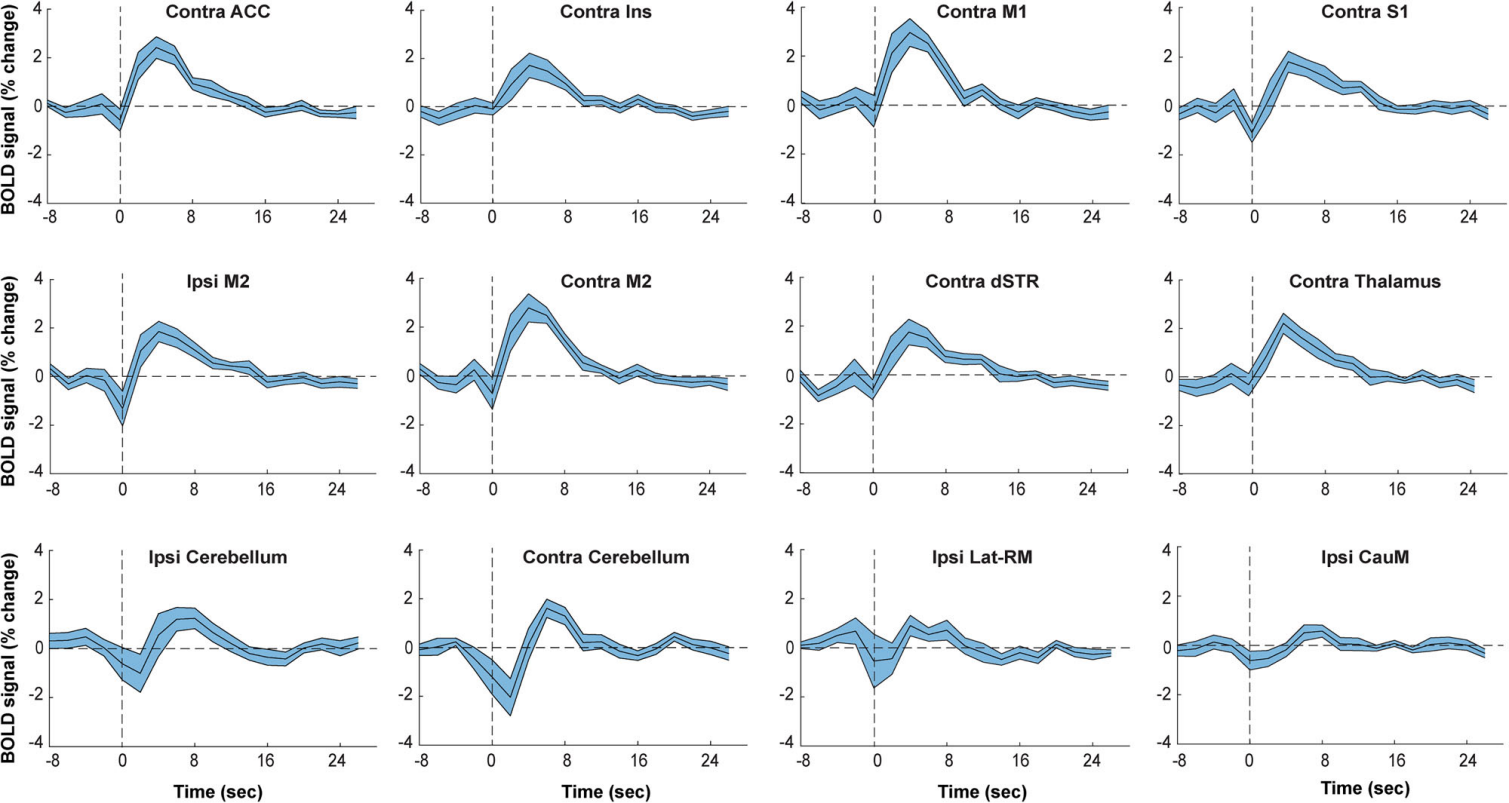
Hemodynamic response profiles to forepaw press averaged across animals in key cortical, subcortical, and brainstem regions. Graphs show the average percentage change in BOLD signal over time, averaged across all successful presses for each animal, with time zero marking the onset of each forepaw press. The profiles exhibit the expected BOLD hemodynamic response, characterized by an initial dip and a response delay of ∼2 s, followed by a time-to-peak of 4–8 s. The largest BOLD response compared with baseline was observed in the contralateral M1. The solid line and blue area represent the mean ± SEM across all animals. Abbreviations are as in Figure 3. Hemodynamic response profiles for a representative animal are shown in Extended Data Figure 6-1.

To investigate temporal response dynamics, we calculated the average hemodynamic response during the forepaw force control task. Extended Data Figure 6-1 shows hemodynamic responses in a representative animal, illustrating average BOLD signal changes in key brain regions time-locked to forepaw press onset. Figure 6 presents the mean percent BOLD signal change across animals, with time zero corresponding to press onset. In contralateral sensorimotor cortical regions (M1, M2, S1, insular cortex, and ACC), BOLD signal increased by ∼2–3%, peaking ∼4 s after press onset. Contralateral subcortical regions, including the dSTR, THL, and CB, also exhibited robust task-related responses. Significant activation was additionally observed in ipsilateral regions, including M2, CB, Lat-RM, and CauM. Notably, responses in the cerebellum and CauM peaked later (∼6 s) compared with other regions. The largest BOLD response compared with baseline was observed in the contralateral M1 (mean 2.96% change). The ipsilateral M2, contralateral S1, and contralateral cerebellum demonstrated the earliest BOLD response change (initial dip) at time zero.

10.1523/ENEURO.0106-26.2026.f6-1 Figure 6-1 **Hemodynamic response profiles to forepaw press in a representative animal across cortical, subcortical, and brainstem regions.** Graphs show the percentage change in BOLD signal over time for a single animal, with time zero marking the onset of each forepaw press. The profiles illustrate characteristic hemodynamic responses, with an initial dip followed by a delayed positive peak occurring approximately 4–8 seconds post-press. Distinct response amplitudes and timings were observed across regions. Abbreviations are as in Figure 4. Download Figure 6-1, TIF file.

## Discussion

Building on our previously established awake mouse fMRI framework (Jindal et al., 2026), we implemented a forepaw force control task to characterize brain-wide activation associated with forelimb force while simultaneously recording applied force. The MR-compatible apparatus enables head fixation and behavioral shaping, allowing mice to perform unilateral forepaw presses on a force sensor for water reward within the scanner environment. To our knowledge, this is the first study to combine awake fMRI with a forepaw force control task in mice, revealing a widespread network of cortical and subcortical regions involved in forelimb force control. Using this approach, we identified force-related activation in sensorimotor cortical areas, striatum, thalamus, cerebellum, and medulla, consistent with findings from human fMRI studies of hand force control (Tanaka et al., 2009; Wasson et al., 2010; Spraker et al., 2012; Chu et al., 2020). This framework enables direct cross-species comparison of brain activity during controlled force tasks.

We further developed a sensitive MR-compatible force sensor to quantify forepaw press strength and deliver reward in real time. Behavioral results showed that mice acquired goal-directed forelimb pressing, progressively refining force magnitude toward the reward threshold and reducing force variability (Fig. 2). Performance improvements primarily reflected enhanced force control rather than temporal adaptation, as animals did not fully align presses with the imposed ITI (Extended Data Fig. 2-1). Instead, the ITI served to maintain engagement and prevent continuous pressing, rather than enforce temporal precision. Accordingly, the task is best described as a mixed task involving force control, reward, and task engagement rather than a pure force control task.

Most prior rodent fMRI studies have been conducted under anesthesia, which alters neural activity and connectivity (Bukhari et al., 2017; Tsurugizawa and Yoshimaru, 2021) and limits comparison with human studies (Mandino et al., 2024). Although the use of awake fMRI has increased, most paradigms focus on simple operant behaviors (Chen et al., 2020) or sensory stimulation (Han et al., 2019). Only one prior study examined awake fMRI during a motor task, reporting activation during lever pressing (Fonseca et al., 2022). However, that task was not force-controlled and involved limited sampling. In contrast, our paradigm enables continuous measurement of force and brain-wide activity during a controlled motor task.

Mean head displacement across all animals and directions remained below half the voxel dimension, supporting reliable estimation of task-related activation with minimal motion artifacts. The combination of head fixation and extensive habituation to the MRI environment before imaging contributed to the low levels of motion observed during scanning. Residual motion effects were accounted for by including six rigid-body motion parameters (three translations, three rotations) as nuisance regressors in the GLM. Volumes identified as motion outliers were additionally included as separate regressors to further mitigate their effects. Representative EPI images before and after motion correction are shown in Movie 2. Furthermore, the continuous force regressor, sampled at the midpoint of each TR and complemented by its temporal derivative, captured average force dynamics within each volume, allowing small timing differences between presses and image acquisition to be effectively modeled within the GLM.

The fMRI results associated with the forepaw force showed significant activation clusters in sensorimotor cortical (M1, M2, ACC, RSG, S1), subcortical (dSTR, THL, hypothalamus, hippocampus), and cerebellar regions (Crus I, simple lobule; Fig. 3*A*). Similar to the previous findings in humans (Ehrsson et al., 2001; Kuhtz-Buschbeck et al., 2008; Spraker et al., 2012), we observed significant activations in the primary motor and somatosensory cortex as well as the cingulate motor area. We also observed significant activations in basal ganglia (dSTR), the thalamus, and the cerebellum, consistent with their essential roles in movement planning and error correction (Vaillancourt et al., 2007; Coombes et al., 2011; Tanaka et al., 2014). These findings are also consistent with electrophysiological and lesion studies demonstrating roles of S1 and M1 in force control (Hays et al., 2013; Mathis et al., 2017). Sensorimotor cortical areas showed a modest contralateral bias relative to the forepaw press, while bilateral limb involvement during the task likely contributed to the observed bilateral cortical activation.

We observed significant activations associated with the water reward delivery in a network of areas including the ventral striatum, second somatosensory cortex, insular cortex, thalamus, septal nucleus, piriform cortex, and sublenticular extended amygdala. These regions are consistent with known circuits involved in reward processing, motivation, and associative learning (Rolls, 1972; Truchet et al., 2002; Haber, 2011; Matsuyama et al., 2011; Cox and Witten, 2019; Midroit et al., 2021; Smith and Torregrossa, 2021; Sniffen et al., 2024). Notably, we did not detect activation in regions such as the hippocampus, nucleus accumbens, and prefrontal cortex. This may reflect a limitation of the experimental design, as sensor pressing and reward delivery were temporally correlated, reducing the ability to dissociate motor and reward-related signals within the GLM. Future studies incorporating jittered or delayed reward delivery could improve isolation of reward-specific activity.

DeepLabCut-based kinematic analysis provided behavioral context for the observed brain activations (Fig. 4). Tracking of the ipsilateral forepaw, elbow, and shoulder revealed activation patterns closely correlated with applied force. The strong spatial overlap between forepaw-related and force-dependent activation suggests that motor output and kinematic control rely on overlapping cortical and subcortical networks. These findings highlight the coupling between force modulation and movement kinematics and demonstrate the sensitivity of the task and analysis framework in capturing naturalistic movement features. However, force production in the current task is inherently associated with movement execution and may also be accompanied by anticipation and preparatory activity. Therefore, the observed activation patterns should not be interpreted as reflecting force control alone.

The distinction between rewarded and nonrewarded presses highlights the influence of reward on motor and associative networks (Extended Data Fig. 3-1). Rewarded trials showed widespread cortical–subcortical activation, reflecting integration of motor execution with reward processing. In contrast, nonrewarded presses exhibited reduced and more spatially confined activation, potentially reflecting lower motivation and sensory engagement. However, this reduction may also be influenced by the limited number of nonrewarded events, reducing statistical power. As the experimental design was not optimized to directly compare rewarded and nonrewarded conditions, these findings should be interpreted with caution. Future studies with balanced trial counts across conditions will be necessary to enable a more rigorous comparison. Additionally, the relatively small number of female mice included in the study limited our ability to adequately assess potential sex-specific differences in behavioral performance and task-related activation patterns.

In order to investigate the contribution of individual brain areas in forepaw force control, we quantified task-related activation time-locked to force presses across sensorimotor regions (Fig. 5) and observed significant activation in brainstem regions, including ipsilateral Lat-RM and CauM. In a recent study examining functional connectivity between cortical and medullary regions in humans and mice, we identified a conserved corticomedullary network involving Lat-RM and CauM that supports forelimb movement control across species (Jindal et al., 2026). Prior studies show that Lat-RM contributes to forelimb control, that MRM contributes to head movements, and that CauM receives Lat-RM input and projects to the spinal cord to mediate skilled forelimb actions (Esposito et al., 2014; Ruder and Arber, 2019; Ruder et al., 2021; Arber and Costa, 2022; Yang et al., 2023). The observed activation in Lat-RM and CauM aligns with these findings and is consistent with the reaching and force-pressing demands of the task.

To further examine thalamic subregions, we quantified task-related activation in THL–VP, THL–PO, and THL–VAL (Fig. 5*B*). THL–VAL showed the strongest activation with a contralateral bias, consistent with its role in sensorimotor integration and goal-directed movement (Catanese and Jaeger, 2021), and its connectivity with cerebellum, striatum, and motor cortex (Tlamsa and Brumberg, 2010). THL–VP, a primary relay for proprioceptive input, and THL–PO, involved in thalamocortical sensorimotor integration (Audette et al., 2019; Vahdat et al., 2021; La Terra et al., 2022), were also engaged during task performance.

Analysis of hemodynamic responses in key brain regions revealed a consistent initial dip in the BOLD signal at the onset of forepaw presses (Fig. 6). This response was most pronounced in ipsilateral M2, contralateral S1, and contralateral cerebellum, suggesting early sensorimotor processing associated with force control. It is unlikely to reflect motion artifacts, as it was absent in regions such as contralateral M1, thalamus, and insula. Given that such initial dips are often not observed under anesthesia (Lindauer et al., 2001; Dunn et al., 2005; Ma et al., 2016), this feature may reflect a characteristic of the awake physiological state.

The contralateral thalamus and dSTR exhibited rapid increases in BOLD signal within ∼2 s of press onset (∼2–3%), consistent with their roles in sensorimotor integration and motor execution, as well as action selection, motivation, and reward processing (Cox and Witten, 2019; La Terra et al., 2022). Similarly, contralateral M1, M2, and ACC showed robust increases in activation time-locked to press onset, underscoring their involvement in movement initiation. In contrast, cerebellar regions and CauM exhibited delayed responses relative to press onset. However, these timing differences should be interpreted cautiously, as regional variations in neurovascular coupling may influence the shape and timing of the BOLD response. The earlier cortical responses relative to cerebellar and medullary regions may reflect differences in neurovascular coupling and regional hemodynamic timing (Devonshire et al., 2012; Shaw et al., 2021). We also observed significant task-related activation in ipsilateral M2, consistent with its role in flexible action selection and the integration of sensory history (Wang et al., 2020).

Weakness and reduced grip strength are common consequences of stroke and movement disorders. Our behaving fMRI framework enables investigation of the neural basis of force deficits and recovery. Combined with mouse models of neurological injury, it may provide insights relevant to improving motor recovery.

## Synthesis

Reviewing Editor: Silvia Pagliardini, University of Alberta Decisions are customarily a result of the Reviewing Editor and the peer reviewers coming together and discussing their recommendations until a consensus is reached. When revisions are invited, a fact-based synthesis statement explaining their decision and outlining what is needed to prepare a revision will be listed below. The following reviewer(s) agreed to reveal their identity: Rupesh Chillale. Note: If this manuscript was transferred from JNeurosci and a decision was made to accept the manuscript without peer review, a synthesis may not be available.

This study applies a novel analytical framework to data previously published study , Jindal et al. (2026), PNAS. The key finding is the identification of activation maps associated with distinct task parameters during the forepaw force task. The incorporation of DeepLabCut strengthens the analysis by enabling active limb tracking alongside representative force-map outputs during task performance. Overall, this represents a strong example of data reuse and advanced secondary analysis of an existing dataset.

I have several comments/questions for the authors:

1.Figures 1A and 1B (schematics) appear to be reused from the group's earlier publication in PNAS (2026). Please clarify whether appropriate permissions were obtained for reuse, if required. it would also be useful to explicitly mention which animals, behavioral analysis and imaging runs overlaps with the prior study. Teasing apart the novelty, the authors should mention which analyses and results are entirely new in the manuscript. This is important because the current manuscript makes strong claims about the brainstem and corticomedullary involvement

2.A few studies have reported differences in average grip force between male and female mice. This study uses only ~20% female adult C57BL/6 mouse mice data. Do the authors believe the findings are broadly generalizable, or are they more representative of adult male C57BL/6 mice? Additionally, were any sex-dependent differences observed in the activation maps or behavioral parameters?

3. The central claim is that the reported activations relate to forepaw force control. However, the force signal is likely correlated with several other variables: forepaw displacement, elbow/shoulder movement, reward delivery, licking, arousal, and motivation. The authors do include reward regressors and kinematic GLMs, but the separability of these regressors remains unclear. I would advise adding a panel to show the percentage of variance explained by each regressor. It would be difficult to disentangle the contribution of each of the variables since they may be correlated to each other. A collinearity diagnostic analysis should be added to the GLM.

4.The authors state that average motion during functional scans was minimal and provide a supplementary video in support. The manuscript cites Fonseca et al., which reported large-amplitude brain signal fluctuations associated with licking behavior and used LASSO regression to predict and remove motion-related artifacts. Did the authors observe similar artifacts associated with forelimb or licking events in the current dataset? If not, do the authors believe the behavioral design or restraint strategy mitigated these effects?

5. The fMRI activity in distributed network could be due to the motor movement of the paw. To reach for the conclusion of motor control, what type of controls were used? How the interpretation be restricted to only control not for other motor related features, such as anticipation and motor preparatory activity.

6. Reduction in the force to 0.3 N can show the movement control, however, figure 1A supplementary shows that the number of presses is not changed between first 3 and the last 3 days. Does this reflect no learning?

7.The group-level activation maps shown in Figure 3A do not appear to show force-associated activation in the medulla. Similarly, Figure 4 does not show activation maps associated with forepaw, elbow, or shoulder displacement during task performance. However, Figure 5C demonstrates responses in the brainstem, particularly in CauM. Please clarify how these results were generated and why these regions are not represented in the earlier activation maps.

8.On page 12, second paragraph, last line, the authors mention a "modest contralateral bias in sensorimotor cortices." The authors should refer readers to Figure 5, which appears to support this statement.

9. The behavioral task may index task-engagement more than precise force control. The animals learned to increase rewarded presses and reduce force variability, but they did not consistently withhold presses during the ITI. The manuscript states that learning criteria were based on rewarded presses rather than ITI compliance. This is acceptable, but it changes the interpretation of the task. Please define more clearly whether the task is primarily a force-control task or an operant pressing task, or a mixed task involving force, reward, and engagement. I would advise the authors to include unrewarded presses, total presses, presses during the ITI, and licking/reward collection behavior across training and scanning.

10. Does the activity was scaled to the press time and how does that change before the press? This question is important because between ITI, whether the animal pressed and the activity was carryforward to the next press. This is not clear from the methods section.

11. We know from the Musall et al., 2019 that the mouse cortex shows the activity for the task-irrelevant movements. In that study, they used features of the movement using video recording. If the camara was used during the recording session, how does the activity correlate with the other features of the forcepaw including the force.

12. Hemodynamic response timing should be interpreted cautiously since the cerebellum and caudal medulla peak later than cortical regions, which is interesting, but interpretation is difficult because regional neurovascular coupling can differ substantially across cortex, cerebellum and brainstem. Though the authors acknowledge this change, but some statements still imply sequential processing. I suggest the authors should consider region-specific HRFs or including finite impulse response analyses to support the timing claim.

13. The brainstem results are crucial, especially the Lat-RM and CauM findings. However, only 9 animals had full brainstem coverage, and ROI-level findings appear to be based on smaller masks with modest effects. I would suggest the authors to state clearly in the Results and figure legends that brainstem analyses use n = 9, not n = 15 and provide the number of voxels included in each brainstem ROI and the criteria used for atlas alignment/manual adjustment. Also for statistical clarity, include correction for multiple comparisons across brainstem ROIs.

14. The figures are informative but could use additional clarity. Figure-3 and Figure-4 contain many slices and labels. Please consider adding enlarged panels for key regions. The unit for the bar plot in Fig 1C is mentioned in the text but is not readily clear from the figure. I suggest the authors to include the y-label for the bar plots.

15.In the Discussion, the authors state: "We developed a method for conducting awake mouse fMRI during a forepaw force control task, demonstrating the feasibility of measuring brain-wide activation related to forepaw force while simultaneously recording applied force."

However, based on the manuscript and the authors' prior publication, the experimental method and dataset were previously established, while the present study primarily introduces a new analytical framework and expanded regional characterization of task-related activation. The discussion should therefore more clearly emphasize the methodological advances in analysis, rather than implying that the behavioral-fMRI acquisition framework itself was newly developed in this study.
