# Supplementary material for: Brain-Wide Activations Related to Forepaw Force Control Identified by Behaving Mouse FMRI
Source: eNeuro. 2026 Aug 18;13(8):ENEURO.0106-26.2026. doi: 10.1523/ENEURO.0106-26.2026 (PMC13485463; doi:10.1523/ENEURO.0106-26.2026)
Supplement: Supplementary file 1 [file eneuro-13-ENEURO.0106-26.2026-s006.docx]

|  | ***Forepaw disp.*** | ***Elbow disp.*** | ***Shoulder disp.*** | ***Forepaw disp.*** | ***Elbow disp.*** | ***Shoulder disp.*** |
| --- | --- | --- | --- | --- | --- | --- |
|  | ***Applied Force*** | | | ***Water delivery*** | | |
| ***r (mean ± SEM)*** | *0.15 ± 0.034* | *0.13 ± 0.03* | *0.15 ± 0.03* | *0.10 ± 0.03* | *0.07 ± 0.03* | *0.09 ± 0.03* |
| ***Mean R^2^*** | *0.023* | *0.017* | *0.022* | *0.009* | *0.005* | *0.008* |
| ***p value (t statistics)*** | *0.0013 (4.01)* | *0.0011 (4.11)* | *0.0118 (4.52)* | *0.0029 (3.60)* | *0.0225 (2.56)* | *0.0118 (2.89)* |

**Supplementary Table 2.** Correlation between the applied force (left columns) and water delivery (right columns) time course with movement kinematic time-series (forepaw, elbow, and shoulder displacement) derived from DeepLabCut. Values represent mean correlation coefficients (r ± SEM), mean coefficients of determination (R²), and associated statistical measures across animals. Although several correlations reached statistical significance, the low R² values indicate that force, reward, and kinematic regressors shared relatively little variance and captured distinct aspects of task performance.
