# Supplementary material for: Brain-Wide Activations Related to Forepaw Force Control Identified by Behaving Mouse FMRI
Source: eNeuro. 2026 Aug 18;13(8):ENEURO.0106-26.2026. doi: 10.1523/ENEURO.0106-26.2026 (PMC13485463; doi:10.1523/ENEURO.0106-26.2026)
Supplement: Supplementary file 2 [file eneuro-13-ENEURO.0106-26.2026-s007.docx]

| **Average Absolute Correlation Value** | | | | | | | |
| --- | --- | --- | --- | --- | --- | --- | --- |
| **Forepaw** | |  | **Elbow** | |  | **Shoulder** | |
|  |  |  |  |  |  |  |  |
| **M01** | 0.04926224 |  | **M01** | -0.040708001 |  | **M01** | 0.13875655 |
| **M02** | 0.160183733 |  | **M02** | 0.070780417 |  | **M02** | 0.07961121 |
| **M03** | -0.011629367 |  | **M03** | 0.00584701 |  | **M03** | 0.0281576 |
| **M04** | 0.062043555 |  | **M04** | -0.130915781 |  | **M04** | -0.07997285 |
| **M05** | -0.095242147 |  | **M05** | -0.083813496 |  | **M05** | -0.05054249 |
| **M06** | -0.118363193 |  | **M06** | -0.135046374 |  | **M06** | -0.15288259 |
| **M07** | 0.037262019 |  | **M07** | 0.012420601 |  | **M07** | 0.03738854 |
| **M08** | 0.004306671 |  | **M08** | 0.102349 |  | **M08** | 0.05221779 |
| **M09** | -0.031042965 |  | **M09** | -0.110436971 |  | **M09** | -0.09780596 |
| **M10** | 0.100355531 |  | **M10** | 0.148894063 |  | **M10** | 0.11220431 |
| **M11** | -0.065897345 |  | **M11** | -0.098075072 |  | **M11** | -0.11028704 |
| **M12** | 0.204926962 |  | **M12** | -0.008632708 |  | **M12** | 0.02537877 |
| **M13** | 0.170892839 |  | **M13** | 0.050839295 |  | **M13** | 0.13653903 |
| **M14** | 0.090565847 |  | **M14** | -0.045427164 |  | **M14** | -0.00991993 |
| **M15** | 0.108919478 |  | **M15** | -0.102009311 |  | **M15** | -0.07673788 |
| **Statistics** | | | | | | | |
| **Mean ± SEM** | 0.0444 ± 0.0252 |  | **Mean ± SEM** | -0.0243 ± 0.0229 |  | **Mean ± SEM** | 0.0021 ± 0.0241 |
| **pvalue** | 0.0995 |  | **pvalue** | 0.3076 |  | **pvalue** | 0.9306 |
| **tstat** | 1.7645 |  | **tstat** | -1.0588 |  | **tstat** | 0.0866 |
